# Deep Learning Wavefront Sensing from Object Scene for Directed Energy HEL Systems

**DOI:** 10.3390/s26010268

**Published:** 2026-01-01

**Authors:** Leonardo Herrera, Nicholas Messina, Brij N. Agrawal

**Affiliations:** Department of Mechanical and Aerospace Engineering, Naval Postgraduate School, 1 University Circle, Monterey, CA 93943, USA

**Keywords:** deep learning, convolutional neural networks, wavefront error, shack-hartmann sensor, adaptive optics, zernike coefficients

## Abstract

Atmospheric turbulence significantly degrades the performance of High Energy Laser (HEL) systems by distorting the laser wavefront as it propagates through the atmosphere. Conventional correction techniques rely on Adaptive Optics (AO), which preserve beam quality at the object. However, AO systems require wavefront sensors, such as Shack–Hartmann, and a reference beam, increasing system complexity and cost. This work presents a Deep Learning (DL)-based wavefront sensing approach that operates directly on scene imagery, thereby eliminating the need for dedicated wavefront sensors and a reference beam. A DL model was trained to predict wavefront distortions, represented by Zernike coefficients, from aberrated imagery of the Reaper Unmanned Aerial Vehicle (UAV). Reaper imagery utilized in training was aberrated at different levels of turbulence, $D/r_{0}$, with D=30 cm being the aperture diameter of a telescope capturing the object scene and $r_{0}=3,\;5,\;7$ cm the Fried parameter that defines weak turbulence for higher values and strong turbulence for lower values. The proposed model, trained across all these turbulence levels, outperformed models trained on a single level by providing superior accuracy and offering practical advantages for deployment. The model also demonstrated strong generalization capabilities for two practical scenarios: (a) Reaper imagery with turbulence levels beyond the training range, and (b) Mongoose UAV imagery not included in the training set. The model predicts turbulence accurately in both cases. The results confirm that if the model is trained for a UAV model for a certain turbulence level, it provides accurate predictions for turbulence levels outside its training range and for other UAV aberrated images.

## 1. Introduction

Adaptive optics (AO), initially developed to improve the imaging performance of astronomical telescopes [1] and more recently applied to directed energy [2,3,4], is a technology that employs a wavefront sensor, an adaptive optical element, a dedicated control computer for real-time wavefront corrections, and a reference point source. The wavefront sensor measures aberrations in the incoming light from a reference point source, such as a natural guide star or laser beacon. As this reference light propagates through the atmosphere or other optical media, it accumulates phase distortions. These distortions, in addition to other aberrations that arise from imperfections in optical components that receive the wavefront and from a moving or vibrating platform on which the optical components are located produce an aberrated wavefront that is measured by the wavefront sensor. The control computer processes the wavefront data and computes the necessary corrections, which are then executed by the deformable mirror to compensate for the distortions. In High Energy Laser (HEL) applications, the outgoing laser beam is deliberately pre-distorted at the deformable mirror such that, after propagating through atmospheric turbulence, the imposed phase aberrations are compensated, enabling precise focusing on the object.

Several wavefront sensing techniques are available [5,6,7], with the Shack–Hartmann (SH) sensor being the most widely used. However, its operation requires precise alignment, is highly sensitive to mechanical vibrations, and entails significant costs due to the need for dedicated optical and imaging subsystems. On the other side, the beacon typically employed as a reference point source is not only complex but also suffers from signal attenuation and reduced return flux, particularly over extended propagation paths, which compromise measurement reliability. Therefore, if turbulence can be predicted directly from the object image—eliminating the need for both the wavefront sensor and reference point source—HEL systems will be greatly simplified and their performance enhanced.

Deep learning (DL) methods, based on Convolutional Neural Networks (CNNs) and more recently Vision Transformers (ViT), provide powerful tools for processing and analyzing image data. Wavefront sensing methods using CNNs [8,9,10,11,12,13,14,15,16,17,18] and ViTs [19,20] have been actively studied in the literature. DL-based wavefront sensing models typically operate on two types of imagery [16]: point-source and scene-based. For point-source methods, a reference beam is artificially created next to the object of interest if a natural bright star is not available. In contrast, scene-based wavefront sensing offers a more practical solution by eliminating the need for a reference point source. Wavefront sensing from object scene imagery has been studied in several works [9,13,16,17,18], demonstrating the potential of deep learning for beaconless AO.

While these works highlight CNN-based wavefront sensing from object scene, there has been limited focus on HEL applications. Our focus is in this context, where maintaining beam quality at the object under dynamic atmospheric conditions is essential. Prior studies [21,22] have investigated beaconless AO in directed energy scenarios using scattered light from the object for wavefront prediction, rather than direct object scene imagery. In contrast, our work extends this line of research by leveraging scene-based imagery itself, evaluated on UAV datasets that represent realistic airborne objects in HEL applications. This underlines our contribution on DL generalization to unseen objects and turbulence regimes, which is critical for operational HEL scenarios.

This work proposes a DL wavefront sensing model that operates directly on object scene imagery for HEL applications. The DL model for wavefront sensing was ResNet-18, a variant of ResNet [23]. The model was configured and trained to predict wavefront errors represented by 15 Zernike coefficients from aberrated Reaper Unmanned Aerial Vehicle (UAV) imagery. The selected Zernike coefficients were the ones after piston, tip, and tilt as these can be corrected by fast steering mirror. Training imagery consisted of aberrated Reaper UAV images at various turbulence levels, $D/r_{0}$, where D=30 cm is the telescope’s aperture diameter capturing the object scene and $r_{0}=3,\;5,\;7$ cm is the Fried parameter. Higher values of Fried parameter indicate weaker turbulence and lower values indicate stronger turbulence. Imagery labels consisted of Zernike coefficients characterizing image aberrations and were generated based on the Fried parameter.

To enrich the dataset and consequently the model performance, each aberrated image was augmented with two additional versions created by applying defocus levels of +10 and −5. These three images (original aberrated, +10 defocus, and −5 defocus) were stacked to form a single Red, Green, Blue (RGB) input, while the 15 Zernike coefficients characterizing image aberrations served as the labels for a supervised learning. This strategy allowed the model to better capture and learn the intricate characteristics of complex wavefronts.

The proposed model was compared against independent models, each trained on specific turbulence values of $r_{0}=3,\;4,\;5,\;6,\;7$ cm. The superiority against independent models was concluded and the model generalization was conducted to predict on data that differ from the training set. As mentioned, our contribution is on DL generalization to unseen objects and turbulence regimes, which is critical for operational HEL scenarios. To assess this generalization, we conducted two practical tests using unseen data: (a) aberrated Reaper imagery with turbulence levels of $r_{0}=4,\;6,\;8,\;9,\;10$ cm—values outside its training range—and (b) aberrated Mongoose UAV imagery not included in the training dataset. Quantitative results confirm that this model is both superior and more practical than the independent models, and that it generalizes effectively to unseen test data.

## 2. Preliminaries

### 2.1. Wavefront Sensing

Wavefront error can be represented using two different approaches: a zonal method and a modal method. A zonal method represents the wavefront error using the phase values at predefined grid points. The modal method represents the wavefront error as a linear combination of Zernike polynomial modal bases such that

(1) $$W\left(x,\;y\right)=\sum_{j=1}^{m}a_{j}Z_{j}\left(x,\;y\right)$$

where, ϕ(x, y) is the phase value of the (x, y)th location, $Z_{j}\left(x,\;y\right)$ is the *j*th Zernike polynomial evaluated at the (x, y)th location, $a_{j}$ is the coefficient for the *j*th Zernike polynomial, and *m* is the number of Zernike polynomials considered in the modal representation of a wavefront. In this paper, the modal method using Zernike polynomials is used to represent the wavefront predicted from the imagery data.

### 2.2. Deep Learning Wavefront Sensing

When the wavefront is represented using Zernike coefficients, the DL problem becomes a regression problem where the output of the DL model is a set of coefficients for the corresponding Zernike polynomials. Deep neural networks using CNN architectures are commonly used for imagery data analysis applications such as object detection and object classification problems, as well as the regression problem considered in this paper. CNNs include the feature extractor network, which employs three types of operations on the data: convolution, Rectified Linear Unit (ReLU) as an activation function, and pooling. The extracted feature map is used in the prediction network to predict the output, which is the modal representation of the wavefront error in our case.

ResNet, introduced in [23], is utilized as the model in this research. ResNet revolutionized deep learning by addressing the degradation problem in very deep networks. Empirically, ResNet-18 has provided superior results on our datasets compared to other CNNs such as GoogLeNet and Xception, while maintaining computational efficiency. Moreover, ResNet was chosen over more modern architectures such as ViTs, since CNNs offer faster inference than ViTs. This property makes ResNet particularly suitable for real-time applications, where optical turbulence changes rapidly, and prediction speed is critical.

The core idea is residual learning, where the desired mapping H(x) is reformulated as: H(x)=F(x)+x, with F(x)=H(x)−x representing the residual function learned by a stack of layers, and *x* is the input that is also passed through a shortcut connection. This design allows the network to learn only the residual difference, which means small changes, rather than the full transformation, making optimization easier. If the optimal mapping is close to identity, then F(x)≈0. This greatly simplifies training because the network only needs to learn minor corrections rather than complex transformations. This structure improves gradient flow because the shortcut provides an unimpeded path for gradients, mitigating vanishing gradient issues. ResNet architectures such as ResNet-18, ResNet-50, and ResNet-152 stack these blocks to enable networks with hundreds of layers without performance degradation.

### 2.3. Data Sets

**Figure 1:**
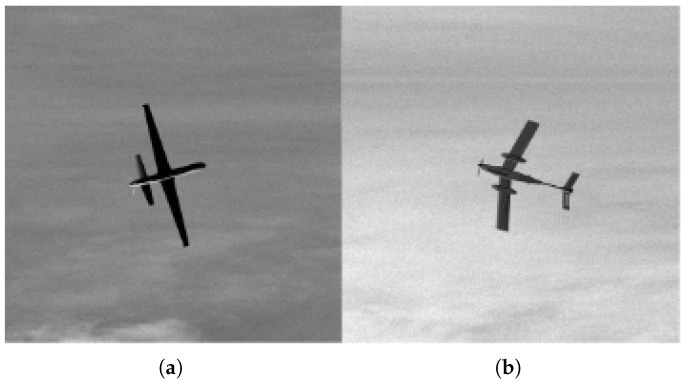
(**a**) Reaper, (**b**) Mongoose.

Two primary datasets were utilized for this research, Reaper and Mongoose. Each of 100,000 simulated images of MQ-9 Reaper and Mongoose drones. The images are gray scale, 256 × 256 pixels, and convey a diverse range of features, including various cloud patterns, different exposure settings, and diverse drone orientations. Figure 1 shows a sample of Reaper and Mongoose.

**Figure 2:**
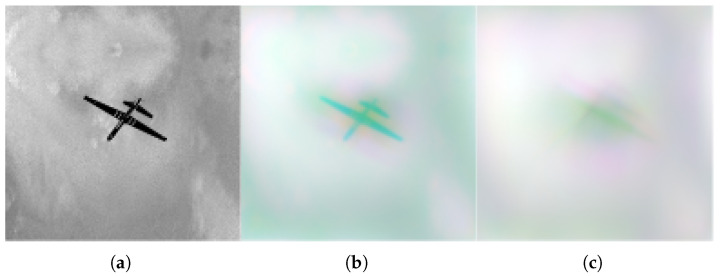
(**a**) Clean Reaper, (**b**) Reaper under weak turbulence, (**c**) Reaper under strong turbulence.

Aberrated datasets were created from the primary datasets at different turbulence levels. All turbulence values were based on the Fried parameter $r_{0}$ with a research range of 3 to 10 cm. This range exhibits the highest turbulence values at 3 cm and then transitions to slightly lower turbulence at 10 cm. Each turbulence value, ranging from 3 cm to 10 cm, was utilized to create 100,000 images at each value: 3, 4, 5, 6, 7, 8, 9, and 10 cm. Figure 2 presents a sample of a clean image from primary datasets next to its blurred versions.

To add turbulence to a clean image, a Zernike polynomia based on $r_{0}$ was applied to the clean image. In total, the first 18 Zernike coefficients were chosen. The first three coefficients were omitted, as they represent only the piston, tip, and tilt. Omitting these values ensures that the model is focused on learning the higher-order and more complex aspects of each aberration. The wavefront was represented by 15 Zernike coefficients (modes 4–18) to model turbulence-induced phase aberrations. The point spread function (PSF), which characterizes how a point source is imaged by the system, was computed using Fourier optics from the complex pupil formed by this simulated wavefront. The resulting PSF was then applied to each clean image through convolution. This process was conducted for all images at all 8 different $r_{0}$ values. Two additional PSFs were produced for each image by modifying only the defocus Zernike coefficient (+10 and −5), and each was convolved once with the clean image to yield further aberrated versions. This procedure is summarized step by step below.

1. **Wavefront expansion (turbulence-induced phase).** The turbulence-induced phase distortion across the pupil is represented as a Zernike expansion,
  (2) $$W\left(x,\;y\right)=\sum_{j=4}^{18}a_{j}Z_{j}\left(x,\;y\right),$$
  where $Z_{j}\left(x,\;y\right)$ are the normalized Zernike polynomials and the coefficients $a_{j}$ quantify the strength of each aberration mode. The coefficients are generated as zero-mean Gaussian random variables whose variances follow Noll’s formulation of Kolmogorov turbulence. In practice, each coefficient is obtained by sampling from the distribution
  (3) $$a_{j}∼N\;\left(0,\;\alpha_{j}{\left(\frac{D}{r_{0}}\right)}^{5/3}\right),$$
  where $\alpha_{j}$ are mode-dependent constants and *D* is the aperture diameter. Air turbulence causes fluctuations in the index of refraction, resulting in aberrations in the laser beam. Air turbulence is commonly referred to as $C_{n}^{2}\left(h\right)$, a measure of fluctuation in the reflected index in a plane. $r_{0}$ Fried number is a widely used metric for quantifying the strength of turbulence at a location. Fried number is given as a function of $C_{n}^{2}\left(h\right)$ as follows:
  (4) $$r_{0}={\left[0.423\;k^{2}\int_{0}^{\infty }C_{n}^{2}\left(h\right)\;dh\right]}^{-3/5},\;k=\frac{2\pi }{\lambda }.$$
  If the aperture diameter *D* equals $r_{0}$, then air turbulence does not affect the laser beam. However, if D/r0 is greater than 1, the turbulence will aberrate the laser beam; the higher the number, the higher the aberration. In the present simulation, $r_{0}$ is treated as a user-defined parameter that controls the overall turbulence strength, rather than being computed from a specific $C_{n}^{2}\left(h\right)$ profile. This choice is common in synthetic data generation, as it allows direct control over the severity of the aberrations. The selected value of $r_{0}$ then sets the statistical distribution of the Zernike coefficients through Noll’s variance model. Zernike coefficients are then created in this research by sampling from the distribution (3). More details in [24,25].
2. **Defocus variation.** Two perturbed wavefronts were generated by adjusting only the defocus coefficient,
  (5) $$W_{\mathrm{psf}}\left(x,\;y\right)=W\left(x,\;y\right)+10\;Z_{4}\left(x,\;y\right),$$
  (6) $$W_{\mathrm{psf}1}\left(x,\;y\right)=W\left(x,\;y\right)-5\;Z_{4}\left(x,\;y\right),$$
  corresponding to over-focus and under-focus conditions. This controlled modification of the Zernike defocus term enables manipulation of the simulated focal shift in pixel units.
3. **Aperture (pupil geometry and transmission).** Light transmission is restricted by the circular aperture function,
  (7) $$A\left(x,\;y\right)=\left\{\begin{array}{cc} 1,\; & \sqrt{x^{2}+y^{2}}\leq D/2,\;\\ 0,\; & \mathrm{otherwise}, \end{array}\right.$$
  which defines the pupil geometry.
4. **Complex pupil (amplitude and phase).** The aperture amplitude and the turbulent phase error combine to form the complex pupil function,
  (8) $$P\left(x,\;y\right)=A\left(x,\;y\right)\;\exp\;\left(i\frac{2\pi }{\lambda }W\left(x,\;y\right)\right),$$
  and for the defocus-perturbed cases,
  (9) $$P_{\mathrm{psf}}\left(x,\;y\right)=A\left(x,\;y\right)\;\exp\;\left(i\frac{2\pi }{\lambda }W_{\mathrm{psf}}\left(x,\;y\right)\right),$$
  (10) $$P_{\mathrm{psf}1}\left(x,\;y\right)=A\left(x,\;y\right)\;\exp\;\left(i\frac{2\pi }{\lambda }W_{\mathrm{psf}1}\left(x,\;y\right)\right).$$
5. **Diffraction to the PSF (image-plane blur kernel).** The Fourier transform of the pupil yields the amplitude spread function, whose squared magnitude defines the point spread function (PSF),
  (11) $$\mathrm{PSF}\left(u,\;v\right)={\left|F\{P(x,\;y)\}\right|}^{2},$$
  with analogous definitions for ${\mathrm{PSF}}_{\mathrm{psf}}$ and ${\mathrm{PSF}}_{\mathrm{psf}1}$.
6. **Image formation and RGB stacking.** Each PSF was convolved with the clean image to produce aberrated versions corresponding to different defocus states. These three images were stacked into the RGB channels, providing the network with multi-plane information that improves prediction accuracy compared to single-plane inputs. Each supervised training sample therefore consists of the RGB stack of defocused images together with the 15 Zernike coefficients $\left(a_{4},\;\ldots ,\;a_{18}\right)$.

## 3. Wavefront Sensing from Object Scene

The DL model was trained on a set with distinct levels of turbulence on Reaper images. The three levels were with $r_{0}$ of 3, 5, and 7 cm. Each level with 100,000 images was divided into 90,000 training images and 10,000 validation images, creating a data set of 270,000 images for training. The trained model is referred to as a combined model, as it is trained with data spanning values of $r_{0}$ of 3, 5, and 7 cm to capture multiple regimes of turbulence. The combined model is analyzed under three scenarios: (a) it is compared with independent models that are each trained on a single, specific turbulence level; (b) it is tested on turbulence conditions that fall both between and outside the $r_{0}$ values used during training, to assess generalization; and (c) it is tested on aberrated Mongoose UAV imagery that was not included in the training set, demonstrating generalization on entirely new data.

### 3.1. Comparison with Independent Models and Generalization to Unseen Turbulence

The combined model is compared with independent turbulence-trained models to determine which method is best for turbulence estimation. Five models focused on singular levels of turbulence were trained on 90,000 Reaper data. The turbulence values $r_{0}$ used for image aberration on independent models were 3 cm to 7 cm. The testing results were on Reaper images for combined and independent models. The combined model was tested on 10,000 Reaper images at $r_{0}$ values from 3 cm to 10 cm for generalization on turbulence levels outside its training range, $r_{0}=4,\;6,\;8,\;9,\;10$ cm. In comparison, each model trained on a single turbulence level was tested on 10,000 Reaper images at corresponding $r_{0}$ values from 3 cm to 7 cm.

**Figure 3:**
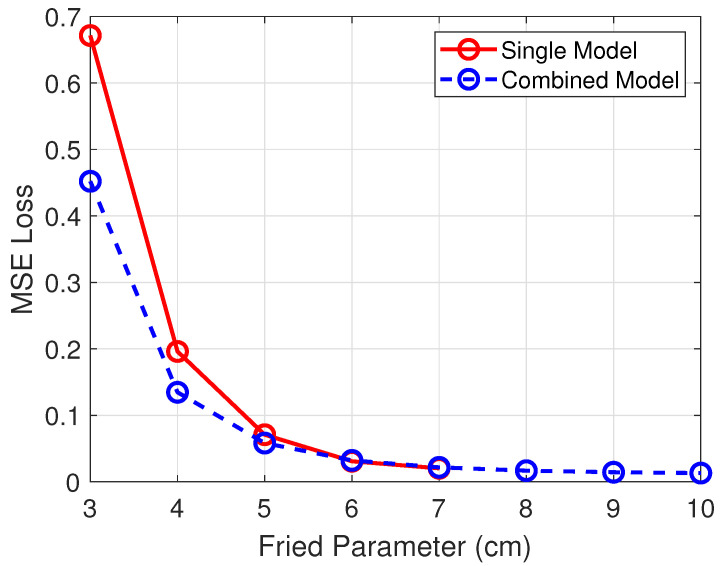
Combined vs. Independent MSE across $r_{0}$.

Results are summarized in Figure 3 for direct comparison. The combined model demonstrated higher accuracy at higher turbulence levels, showing that having more training data is directly related to better performance at high turbulence levels. At lower turbulence levels, the performance of both approaches is similar. In addition, multiple independent models are impractical from an operational point of view. Therefore, the combined model represents the most practical solution and also better performance at higher turbulence levels. From Figure 3 is also observed that the combined model generalizes to aberrated Reaper imagery with turbulence levels outside its training range, $r_{0}=4,\;6,\;8,\;9,\;10$ cm.

**Figure 4:**
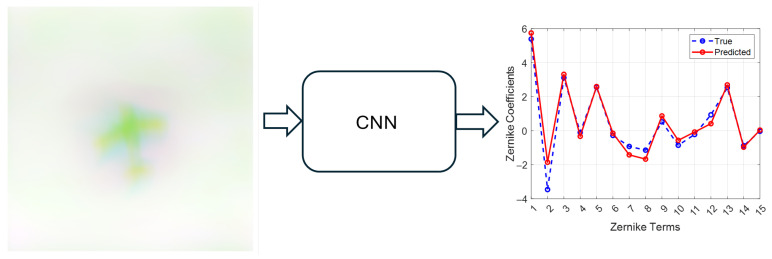
True vs. predicted Zernike, $r_{0}=4$ cm.

**Figure 5:**
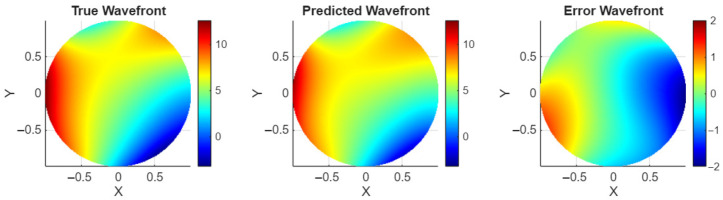
Wavefront results, $r_{0}=4$ cm.

Figure 4 shows the performance of the combined model on predicting Zernike values from a sample image aberrated with $r_{0}$ of 4 cm. The turbulence level corresponding to 4 cm was chosen as an example to represent a high turbulence level at an untrained value. Figure 5 shows the wavefronts determined from the Zernikes in Figure 4 for wavefront visualization. This is a promising result for the combined model, with no noticeable drop in performance at turbulence values beyond those in the trained data.

### 3.2. Model’s Generalization to Different UAV

**Figure 6:**
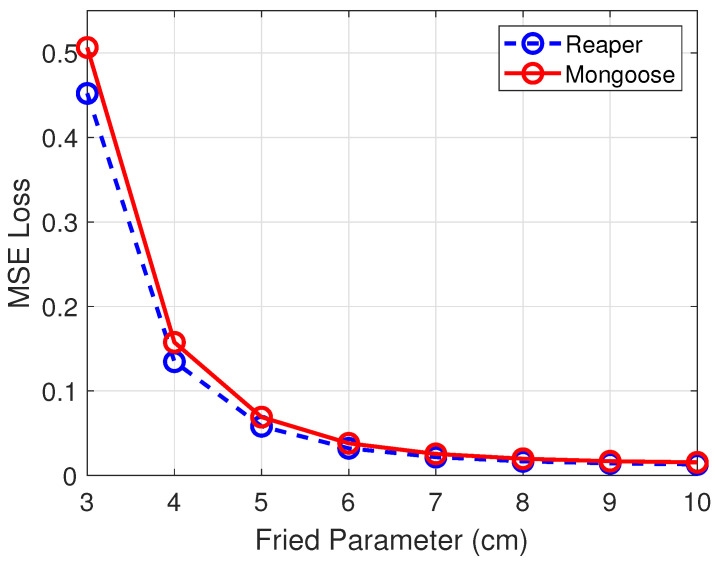
Reaper vs. Mongoose MSE across $r_{0}$.

To further analyze the model’s generalization, the focus shifted to Mongoose drone tests. Figure 6 compares the MSE loss for the testing sets at all turbulence levels between the two drones. Not only did the model follow the same trends, but the error between the two drones was also smaller at lower turbulence levels. Overall, the model performed well on unseen Mongoose data with only a slight drop in accuracy across the turbulence values. This generalization to different UAV shows that the network learned turbulence distortions across the entire image rather than object-specific features because the dataset incorporates varying backgrounds and lighting conditions for each image. The model consistently predicts turbulence despite these variations, which demonstrates that it has learned turbulence itself rather than variations around the objects.

### 3.3. Discussion

While simulation provides a controlled and reproducible environment for initial model development, it cannot fully capture the complexity of real-world atmospheric turbulence and sensor noise. We acknowledge this limitation and emphasize that the present work represents an initial step toward real-time wavefront sensing experimentation—in laboratory turbulence simulators and ultimately in field deployments under real atmospheric conditions.

Despite the simulation, the model demonstrated strong generalization under the conditions under which it was tested. It was able to predict on turbulence and a UAV that were not included in its training set. This generalization was performed under varying image backgrounds and lighting conditions, underscoring the robustness of the model.

Since it is neither feasible nor practical to train models across all turbulence levels or UAV types, demonstrating previous generalization provides evidence of robustness and operational relevance in the HEL context, where maintaining beam quality at the object under dynamic atmospheres is essential.

## 4. Conclusions

Deep learning-based wavefront sensing from object scenes in adaptive optics for HEL systems is a promising approach, as it eliminates the need for both a reference beam and a wavefront sensor. A model trained across multiple turbulence levels outperformed independent models trained on specific turbulence values and offers a more practical solution for real-world deployment. Furthermore, the model demonstrated strong generalization to practical scenarios by accurately predicting turbulence on unseen data with turbulence levels outside its training range and for other UAV aberrated images. This generalization capability is particularly attractive, as it enables accurate turbulence prediction on data for which the model was not explicitly trained in terms of turbulence levels and UAV. Further work is needed to determine the performance for higher turbulence conditions. However, for medium turbulence, deep learning models can be used for predicting turbulence from the object image, resulting in a major simplification of adaptive optics for HEL systems.

## Data Availability

Data available upon request to the corresponding author.

## Abbreviations

The following abbreviations are used in this manuscript:

AO	Adaptive Optics
CNNs	Convolutional Neural Networks
DL	Deep Learning
HEL	High Energy Laser
MSE	Mean Squared Error
PSF	Point Spread Function
RGB	Red, Green, Blue
ReLU	Rectified Linear Unit
SH	Shack–Hartmann
UAV	Unmanned Aerial Vehicle
ViT	Vision Transformer
